# Exploring the Mediating Effect of Physical Activities on Built Environment and Obesity for Elderly People: Evidence From Shanghai, China

**DOI:** 10.3389/fpubh.2022.853292

**Published:** 2022-03-11

**Authors:** Yang Xiao, Sijia Chen, Siyu Miao, Yifan Yu

**Affiliations:** ^1^Department of Urban Planning, College of Architecture and Urban Planning, Tongji University, Shanghai, China; ^2^Beijing Municipal Institute of City Planning and Design, Beijing, China

**Keywords:** elderly people, BMI, physical activity, built environment, mediate effect

## Abstract

There is still a scarcity of literature on the specific mechanisms of the linkage between the built environment and obesity. As a result, this study investigated whether and how physical activities mediate the associations between the objective built environment and the BMI of elderly people. To investigate the effect of the duration and intensity of physical activity on the effect of the built environment, the study made use of the bootstrap method. In general, we discovered that physical activity duration has a huge mediating effect on the elderly people in Shanghai, especially with respect to the density and accessibility of facilities (gyms, parks, fast-food restaurants) that can greatly stimulate physical activity in elderly people to reduce their BMI. There were both direct and indirect effects on their BMI, which means that the health benefits of green spaces for older people may be more complicated than first thought.

## Introduction

According to the World Health Organization's key facts on obesity, worldwide obesity has nearly tripled since 1975. Most of the world's population lives in countries where the mortality rate of overweight people is higher than that of underweight people. Obesity is now so prevalent in the world's population that it is beginning to supplant undernutrition and infectious diseases as the leading cause of illness (1). Many studies have found it to be associated with an increased risk of type 2 diabetes (2), coronary heart disease (3), hypertension (4), respiratory disease (5), and premature death (6). Less than 5% of adults over the age of 65 in the United States and Sweden met physical activity recommendations (7, 8). It is known that the built environment, such as proximal green spaces, recreation facilities, and street design, can reduce the risk of obesity through a change in people's behavior (9–13). The physical characteristics of the urban environment have ever since influenced how city residents live and work, as well as having a direct impact on their mobility and social interactions (14, 15). For example, Colom et al. (16) discovered that older adults who lived in more walkable neighborhoods had higher levels of physical activity and a lower BMI. Hirsch et al. (17), on the other hand, conducted a longitudinal study and discovered that the relationship between BMI and neighborhood walkability is weak in older adults. According to Creatore et al. (13), there is still a lack of evidence that a more walkable urban neighborhood design could increase physical activity, particularly for different built environments and demographic groups of people.

As for the relationship between the built environment and obesity, emerging global evidence has revealed the indirect pathways by which the built environment influences the risk of obesity, but research on the elderly group is still limited, particularly for China. From 1975 to 2014, the number of obese men and women in China surpassed that of the United States, making it the world's first obese country (18). Many studies confirm that the daily lives of the majority of elderly people revolve around their homes (19). Thus, it is critical for local municipalities to provide a health-oriented built environment that encourages physical activity in the prevention of obesity. In this vein, this study would be aware of the specific mechanisms of linkage between the built environment and obesity, and would investigate whether physical activities mediate the associations between the perceived built environment and the BMI of elderly people. Valid samples came from 10 communities in Shanghai's Yangpu district, where 17.3% of the 60-year-old population lived in 2017. We chose 12,780 samples from these 10 communities. We used a statistical estimation method similar to Preacher and Hayes (20) and Wang et al. (21), employing bootstrap mediation analysis to reflect how physical activity time and intensity mediate the health effects of the traditional 5D features on elderly people's BMI, such as density, land-use mix, street design, accessibility to transit, and accessibility to local amenities.

## Literature Review

### Built Environment and Obesity

Numerous studies have established a fundamental consensus that the built environment influences an individual's healthy behavior choices and activity processes, resulting in a variety of health outcomes such as diabetes, depression, cardiovascular disease, and so on. Existing literature indicates that the built environment has a high potential to promote physical activity, which is thought to be a significant deterrent to overweight and obesity (14). People who live in areas with more compact street networks, higher population density, higher walkability, higher land use mixed use, better aesthetics, and less convenience of facilities and side work will walk more (22). For example, Black et al. (23) and Frank et al. (24) confirmed that residents in communities with poor walking environments will have problems such as being overweight or obese, and that land-use mix is the most prevalent built-environment attribute to have an impact on BMI (25, 26). In addition to calorie expenditure, the availability of food stores in the neighborhood may also influence residents' energy use (27). In Salt Lake City, Utah, Xu et al. (28) In Salt Lake City, Utah, Xu et al. (28) found that the number of fast-food restaurants near people's homes was a strong and positive predictor of their weight and obesity.

### Different Pathway From Built Environment to BMI

Given this, the current evidence does not establish a clear relationship between built-environment features and obesity (10, 29), due to the wide range of built-environment measures (10, 14, 30, 31). According to some studies, objective and perceived environments have a different or inverse effect on physical activities (29, 32, 33). According to Gebel et al. (34), those who perceived their neighborhood as less walkable were less active than those who had determined the same objectively. Stowe et al. (31) discovered that neighborhood walkability affects youth BMI differently depending on geographic location, with positive, negative, and no associations found in urban, rural, and peri-urban areas, respectively. Furthermore, the relationship between physical and built environmental variables and obesity varies according to people's sociodemographic status, such as age (29, 35). This is because people's physical functions deteriorate with age, and age-related functional limitations may make overcoming environmental barriers more difficult (36). Clarke and George (37) confirmed that older adults in residential neighborhoods were less able to perform daily utilitarian activities. According to Li et al. (22), an increase in fast-food restaurant density of a standard deviation results in a 7% increase in overweight and obesity rates among 50–75-year-old adults in Portland. The environment appears to play a more important role in the decline in functioning associated with aging; however, older adults have been the least studied age group, with inconsistent results (38, 39). Colom et al. (16) and King et al. (12) discovered that older adults who lived in more walkable neighborhoods had higher levels of physical activity and a lower BMI. However, Berke et al. (40) discovered that low BMI levels were not found in higher walkability neighborhoods, particularly for men. Xiao et al. (41) use a multilevel two-part model to investigate the relationship between the built environment (neighborhood greenness) and whether and how much physical activity occurs; and they only find that eye-level greenness increases the likelihood of total physical activity and active transportation occurring. The built environment has different effects on older people based on what they do for exercise.

Some scholars recently advocated further research into the specific mechanisms of the linkage between the built environment and obesity (29, 42), as the built environment may work to influence overweight through mediators such as different levels of physical activity, travel mode, sedentary behavior, and social capital (16, 21, 26, 43). According to Poortinga (44), the majority of the mediations of PA on neighborhood environment and obesity are limited, implying that there are other unobserved mediating variables that negatively affect health outcomes in highly walkable neighborhoods. In comparison, Wang et al. (21) discovered that physical activities mediated 46.2% of the contribution of the home environment to adolescent BMI in China, and Koohsari et al. (43) confirmed that light and moderate-to-vigorous physical activities partially mediated the relationships between walkable urban design attributes and older adults' BMI in Japan.

To our knowledge, there is little evidence on the indirect pathways through which the built environment influences obesity risk, particularly among the elderly in China. According to statistics, the proportion of Chinese citizens over the age of 60 was 17.3% in 2017. This figure is expected to rise to 39% by 2050, but Shanghai has become an aging city, with 35.2% of its citizens over the age of 60 at the end of 2019. Based on this premise, this study seeks to bridge the existing gap in literature by investigating whether and how physical activities mediate the associations between the objective built environment and the BMI of the elderly. (1) The health effects of the traditional 5D built-environment features, such as density, land use mix, street design, accessibility to transit, and accessibility to local amenities, on the elderly's BMI. (2) The interaction effect of the built environment, physical activity, and BMI in Shanghai's elderly.

## Study Area and Data Sources

**Figure 1:**
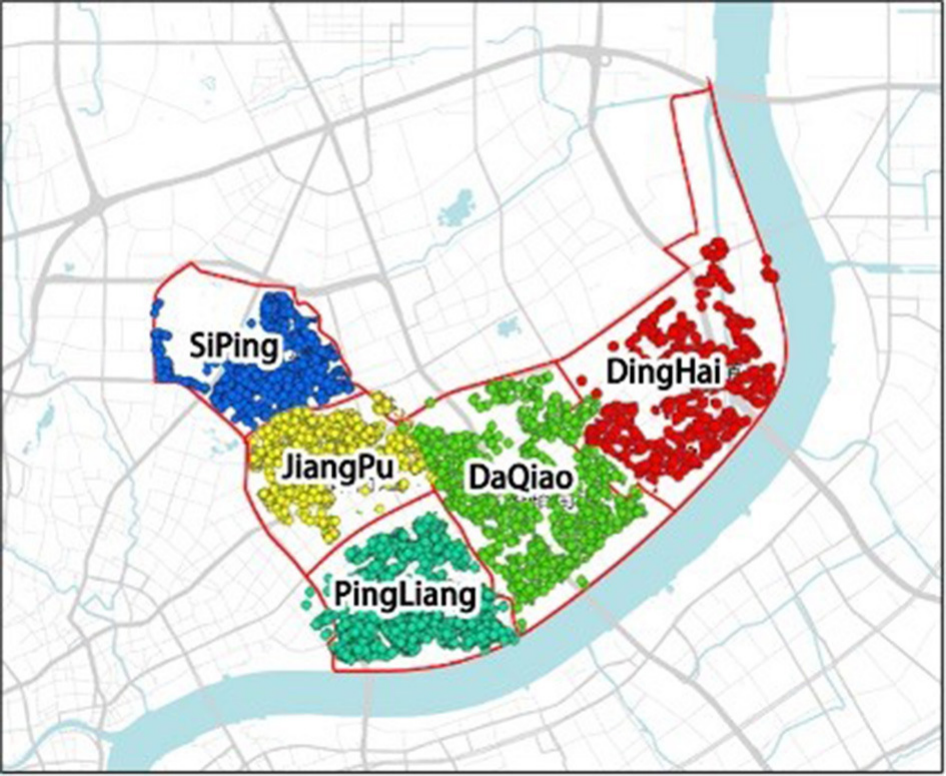
Map of the study area and respondents' locations.

The study used the 2015 Shanghai Yangpu District Diabetes Health Records database to screen out health records of subjects over 60 years old with both BMI and physical activity level records as sample data. Twelve thousand seven hundred eighty valid sample data are present in Figure 1, covering 118 residential communities in five streets of Yangpu District, namely Siping, Jiangpu, Pingliang, Daqiao, and Dinghai.

Table 1 shows the attribute distribution of valid samples. According to the sample's gender, age, self-reported health, diet behaviors, physical activity duration and intensity, and BMI distribution, the number of women in the study sample is slightly more than men, with an average age of 73 years. Over 70% of the elderly are in good health conditions and eat regularly. Regarding their level of exercise, most participants engage in physical activity on a daily basis with varying intensities and duration, in spite of the fact that more than 50% of the elderly are overweight or obese.

**Table 1:** Descriptive statistics for the study variables.

	**Variable**	**Sample**	**Proportion**
Sex	Male	5,476	42.8%
	Female	7,304	57.2%
Age	60–64	1,941	15.2%
	65–69	2,282	17.9%
	70–74	1,722	13.5%
	75–79	1,793	14.0%
	80–84	2,684	21.0%
	Above 85	2,358	18.4%
Diet behaviors	< Three times a day	3,645	28.5%
	=Three times a day	8,676	67.9%
	>Three times a day	459	3.6%
Self-reported	Good health	9,286	72.7%
health	Poor health	3,494	27.3%
Physical activity	<1 h/per day	4,186	32.8%
duration	1–2 h/per day	7,117	55.7%
	>2 h/per day	1,477	11.5%
Physical activity	Light	3,405	26.6%
intense	Moderate	8,689	68%
	Vigorous	686	5.4%
BMI status	Normal (BMI <24 kg/m^2^)	6,616	51.8%
	Overweight (24 ≤ BMI ≤ 27.9 kg/m^2^)	4,843	37.9%
	Obesity (BMI ≥ 28 kg/m^2^)	1,321	10.3%

### Measures

#### Health Outcomes

Since the second half of the nineteenth century, when Nightingale's environmental theory was first proposed, people have recognized the built environment's influence on health outcomes (45). As research advances, more people recognize that the built environment plays a critical role in the quality of medical care and may have an effect on certain health outcomes. Apart from disability, physical impairment, asthma, cardiovascular and cerebrovascular diseases, hypertension, and self-rated health, obesity is one of the most important health outcomes involved. BMI (kg/m^2^) is an internationally recognized standard for determining a person's obesity and health status. We drew on previous research on obesity in the elderly (46, 47), employing Chinese local criteria for obesity and overweight in the elderly. It suggests the BMI classification with cut off points for older adults, BMI <24 is considered normal, between 24 and 27.9 is considered overweight, and more than 28 is considered obese. The subject's BMI, calculated by dividing one's weight in kilograms by one's height in meters squared, as a dependent variable to determine the mechanism and influence relationships between the built environment, physical activity, and BMI, as well as to provide support guidance for the elderly (48).

#### Physical Activity

Physical activity is usually evaluated in terms of duration, frequency, intensity, and type (49). Direct methods of assessing physical activity include self- or interviewer-administered questionnaires, diary annotation, and mechanical or electronic motion sensors such as pedometers, surveillance cameras, self-contained body-action recorders, or remote-reading telemetric devices (50). We use physical activity status (physical activity duration and intensity) as a mediator variable to establish the influence path and calculate the size of the mediating effect. Participants reported the intensity of the activities they engaged in as well as the average number of hours they spent performing the activities per day. Physical activities' intensities were divided into three categories: light, moderate, and vigorous. Light activities included planting flowers and doing housework; moderate activities included jogging/running, dancing, and playing badminton; and vigorous activities included lifting weights and riding. Physical activity durations were divided into <1 h, 1–2 h, and more than 2 h.

#### Built-Environment Characteristics

The built-environment elements that affect health outcomes include the 5D variables of the built environment: density, diversity, design, distance to transit, and destination accessibility (51). The relationship between the built environment, physical activity, and health outcomes can be investigated through a variety of quantitative indicators such as land-use mix, residential density, and street connectivity. The literature discusses the residents' age, occupation, and socioeconomic status (SES), as well as neighborhood safety, food environment, and self-reported health. Certain built-environment elements are specifically designed to influence certain health outcomes. In the field of obesity research, elements such as land-use mix, transport system design, leisure and entertainment facilities, and the food environment are crucial factors. In this study, we screen built-environment variables based on the 5D built-environment framework, as well as examine several related types of physical activity facilities (hospitals, parks, gyms, fast-food restaurants) (see Table 2).

**Table 2:** Sample built-environment characteristics.

**Characteristics**	**Mean**	**SD**
**Density (the number of individuals per hectares)**		
Population density	346	273.78
**Diversity (within 500 m dwelling buffer)**		
Land mix use	0.8	0.13
**Design (within 500 m dwelling buffer)**		
Street density	10	4.41
**Distance to transit (distance in meters from dwelling)**		
Bus stop	173.1	86.43
**Destination accessibility (facility number within 500 m buffer)**		
Hospital	2	1.02
Park	1	1.45
Gymnasium	2	1.17
Fast food restaurant	15	10.71

Population density was operationalized as the number of individuals per hectare. Data on population density was retrieved from the Sixth National Census. The road net density was equal to the ratio of the total length of all roads in a certain calculation area to the total area. The entropy algorithm created by Frank et al. is used to quantify the degree of land-use mix. This approach is notable for its ability to accommodate a wide range of land-use types. The entropy score is equal to 1 if the land use is as diverse as possible, which is the underlying premise of the computation, and the entropy score is 0 when the land usage is as homogeneous as possible (52). The elderly's activity scope and modes differ from those of ordinary adults. We, therefore, choose a 500-m radius buffer zone (approximately a 10-min walking distance) around the sample residence to calculate the number of facilities (hospital, park, gymnasium, fast-food restaurant) to ensure the calculation method of destination accessibility is consistent with the elderly's characteristics. The degree of land-use mix and destination accessibility were calculated by ArcGIS software.


$$\begin{array}{l} \text{Lum }=\;\left(-1\right)\overset{\text{n}}{\underset{\text{i }=\;1}{\sum }}\left(\frac{\text{bi}}{\text{a}}\right)\ln\left(\frac{\text{bi}}{\text{a}}\right) \end{array}$$

### Statistical Method

Following Wang et al. (21), the bootstrap mediation analysis was used to explore the mechanism between built-environment characteristics (X), physical activity (M) and BMI (Y). While assuming that the independent variable X has a direct influence on the dependent variable Y, it is also expected that the mediating variable M has an indirect effect on the dependent variable. One or both effects may be present at the same time, or neither of these outcomes is true. Using physical activity as a mediator variable has become increasingly common, and existing studies have fully proved that different types of physical activities will be affected by different elements of the built environment (53). Moreover, environmental concerns for illness prevention and treatment have received considerable academic interest (11).

There are four common methods to test the mediation effect: stepwise regression, product coefficient Sobel test, coefficient of difference test and Bootstrap method. The most frequently used approach for examining the mediation effect is Baron and Kenny's stepwise regression. Fitting four regression equations reveals the link between the three essential variables; among them, equation ϵ is the error term of the model, and a, b, c, and c', respectively, represent the regression coefficients (54). However, the stepwise regression method has the lowest test effectiveness of the several methods. When the mediating effect is insignificant or a suppression effect exists (54), the stepwise regression method has difficulty detecting it, and may miss certain true mediating effects (55). The bootstrap technique is a more reliable approach compared to other test methods for mediating effects. It is a well-known approach that can be used to substitute the Sobel method and test the product of coefficients directly. Bootstrap sampling is a statistical technique founded on the concept of standard error, wherein a mediating effect is present if the 95% confidence interval does not include the number zero. There is no mediation if the 95% confidence interval contains zero (56).

**Figure 2:**
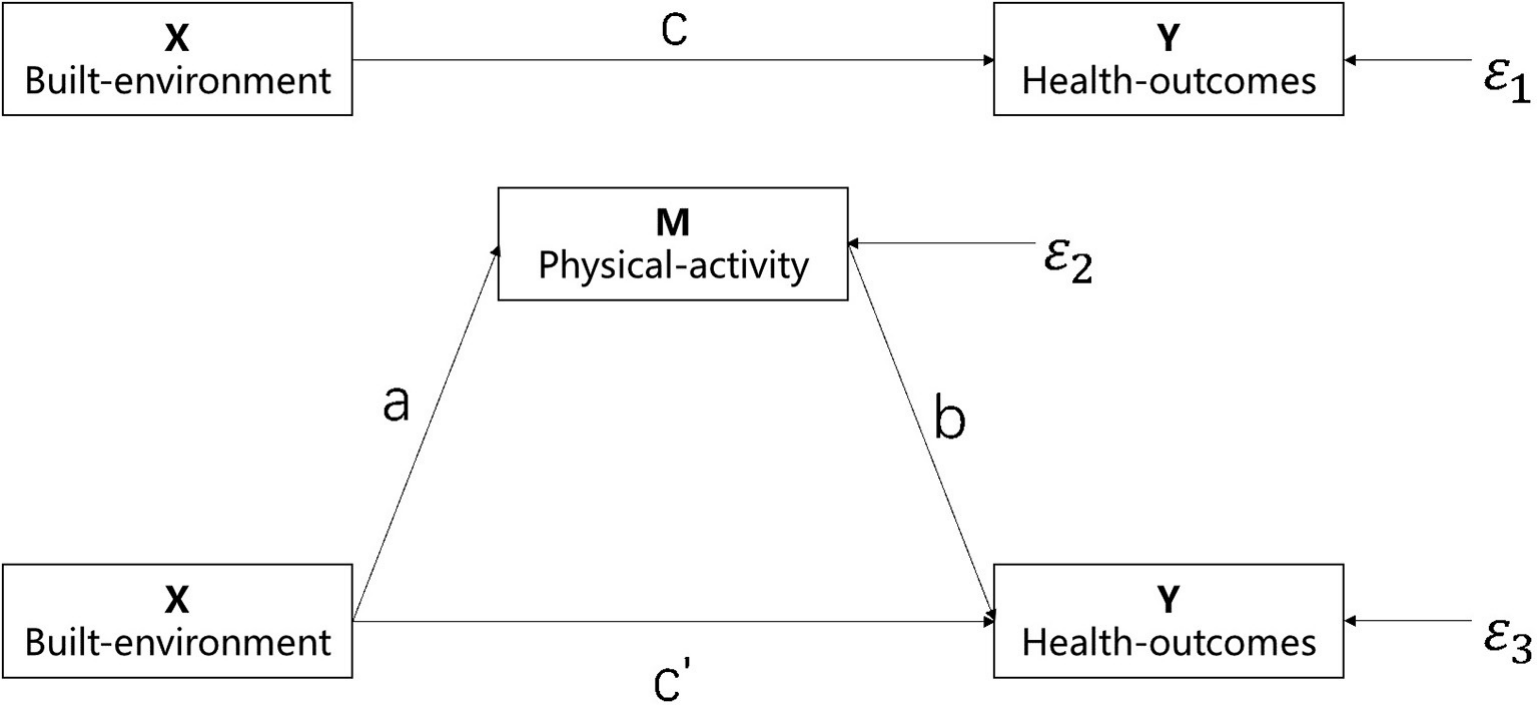
The logical flow of research methods.

Furthermore, independent SPSS entails additional steps for the use of intermediary and adjustment models, and multiple models must be tested in sections only. Hayes addressed this gap by developing a plug-in for intermediary and adjustment effect analysis on the SPSS and SAS-based programs. PROCESS is an SPSS plug-in that implements a deviation-corrected percentile bootstrap method. It enables researchers to conduct direct analysis of mediation and adjustment models. This article uses the bootstrap method to ascertain whether physical activity acts as a moderator between the built environment and health outcomes, as well as the size of the moderate effect. All mediation analyses are controlled for participants' age, gender, self-reported health and diet habit (see Figure 2).

## Empirical Results

**Figure 3:**
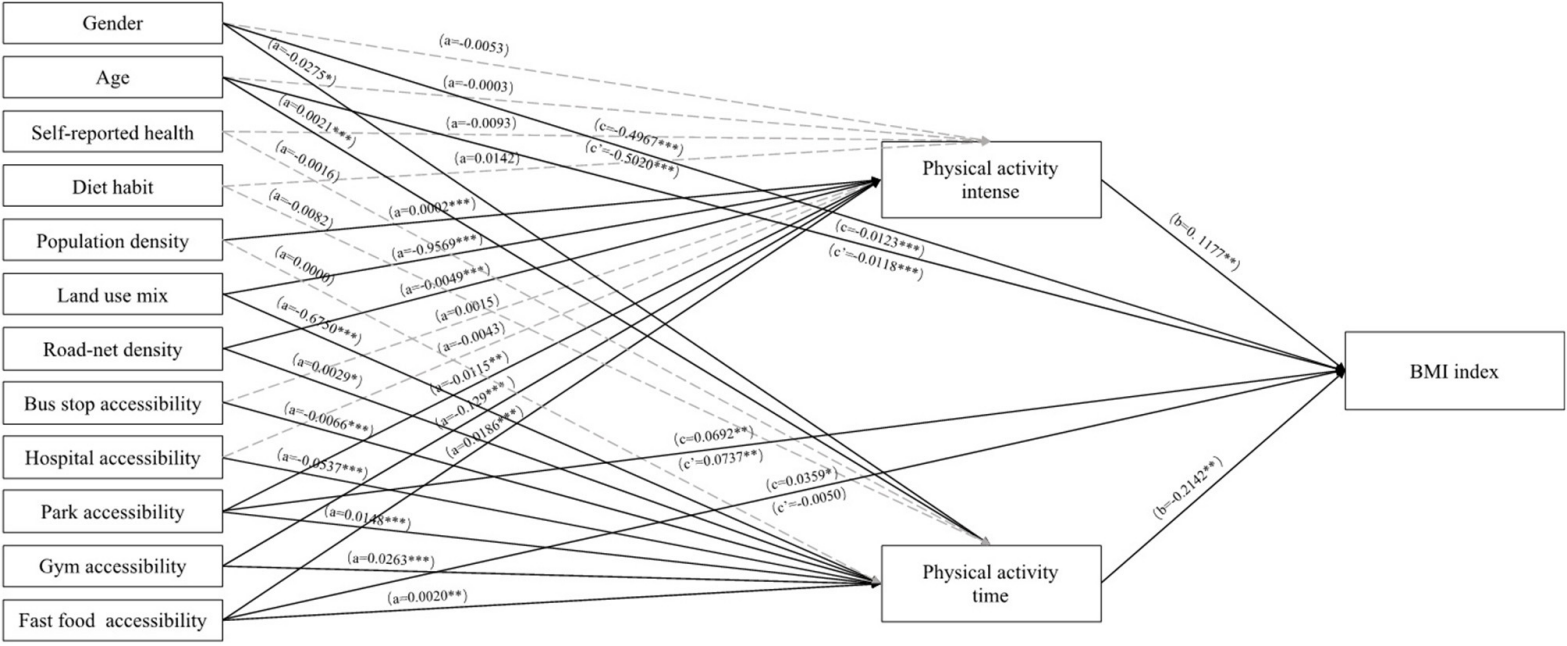
Physical activity mediation model controlled for sex, age, self-reported health, and eating habit. *** *p* < 0.001; ^**^ *p* < 0.01; ^*^ *p* < 0.05.

The bootstrap-mediated analysis model comprises three models: (1) Model 1 used the mediator variable 1, i.e., physical activity intensity, as the dependent variable to examine the effects of relevant factors such as residents' built-environment and socioeconomic characteristics on it; (2) Model 2 used the mediator variable, physical activity time, as the dependent variable; and finally, (3) Model 3 examined the effects of physical activity intensity, physical activity time, community built-environment characteristics, socioeconomic characteristics, and other confounding variables on the residents' BMI. The bootstrap approach was used to examine the direct, indirect, and total influence of built-environment characteristics on BMI, and the proportion and significance of indirect effects via physical activity intensity and time (see Figure 3, Table 3).

**Table 3:** The regression results of three models.

	**Model 1**	**Model 2**	**Model 3**
	**Physical activity intensity**	**Physical activity time**	**BMI index**
**Control variable**			
Gender	−0.0053^a^ (0.0098)	−0.0275^*^ (0.0111)	−0.5020^***^ (0.0585)
Age	−0.0003 (0.0005)	0.0021^***^ (0.0006)	−0.0118^***^ (0.0031)
Self-reported health	−0.0093 (0.0110)	−0.0016 (0.0125)	0.0792 (0.0658)
Diet habit	0.0142 (0.0105)	−0.0082 (0.0118)	0.0882 (0.0625)
**Density**			
Population density	0.0002^***^ (0.0000)	0.0000 (0.0000)	0.0000 (0.0001)
**Diversity**			
Land-use mix	−0.9569^***^ (0.0508)	−0.6750^***^ (0.0573)	0.3841 (0.3081)
**Design**			
Road-net density	−0.0049^***^ (0.0012)	0.0029^*^ (0.0786)	−0.0117 (0.0072)
**Distance to transit**			
Bus stop	0.0015 (0.0010)	−0.0066^***^ (0.0011)	0.0045 (0.0061)
**Destination accessibility**			
Hospital	−0.0043 (0.0050)	−0.0537^***^ (0.0057)	−0.0443 (0.0302)
Park	−0.0115^**^ (0.0036)	0.0148^***^ (0.0041)	0.0737^***^ (0.0216)
Gymnasium	−0.0129^***^ (0.0024)	0.0263^***^ (0.0027)	−0.0044 (0.0144)
Fast-food restaurant	0.0186^***^ (0.0005)	0.0020^**^ (0.0006)	0.0050 (0.0034)
**Physical activity**			
Physical activity intensity	–	–	0.1177^*^ (0.0528)
Physical activity time	–	–	−0.2142^***^ (0.0468)

*** *p < 0.001;*

** *p < 0.01;*

* *p < 0.05*.

a *The coefficients of the model with standard error in parentheses*.

*The significant coefficients are in bold*.

The results of Model 1 and Model 2 indicated that the elderly's physical activity is influenced by all five dimensions of the built environment: density, diversity, design, distance to public transit, and destination accessibility. Among these, land-use mix, road network density, number of parks, gymnasiums, and fast-food restaurants all have an effect on both the intensity and time of physical activity. The more diverse the land use, the lower the residents' physical activity intensity (β = −0.9569, *p* < 0.001) and physical activity time (β = −0.6750, *p* < 0.001). The greater the road network density, park accessibility, and gym accessibility, the lower the intensity but longer the duration of residents' physical activity. This may be because the elderly are more likely to choose low-intensity but prolonged physical activities such as walking when facilities such as road network density, parks, and gyms are increased. As for fast-food restaurants, residents may voluntarily participate in physical exercises of higher-intensity and longer duration to burn excess calories after consuming more fast food (β = 0.0186, *p* < 0.001 and β = 0.0020, *p* < 0.01, respectively). Residents' physical activity intensity was primarily influenced by community population density (β = 0.0002, *p* < 0.001); the higher the community population density, the greater the residents' physical activity intensity. However, community population density had no significant effect on residents' physical activity time. Furthermore, access to public transportation and the number of hospitals had a significant effect on physical activity time (β = −0.0066, *p* < 0.001 and β = −0.0537, *p* < 0.001, respectively). However, they had no significant effect on the intensity of residents' physical activity. Greater distances to the bus stop and more hospitals around the community meant that residents spent less time being physically active. This may be due to the fact that, with an increasing distance to bus stops, the elderly were less willing to walk to their destinations. In terms of control variables, self-rated health and dietary habits had no significant effect on physical activity, although gender and age did, as women had more physical activity time (β = −0.0275, *p* < 0.05). Moreover, the elderly's physical activity time increased with age (β = 0.0021, *p* < 0.001).

Model 3 incorporates the combined effect of factors such as physical activity and built-environment characteristics on the elderly's BMI. Results from this model indicated that physical activity intensity, time, park accessibility, age, and gender, all affected the elderly's BMI. The duration of physical activity has been shown to reduce the elderly's BMI, whereas the intensity of physical activity has been shown to increase the elderly's BMI. This may be because prolonged low-intensity physical activities, such as swimming and jogging, have been shown to promote fat loss and weight loss (57), whereas high-intensity exercises, such as fast running and other intensity training, increase muscle mass, and thus, body weight. In terms of destination accessibility, the more accessible a park is, the higher the residents' BMI. Women have a greater BMI, while older people have a lower BMI.

Using the bootstrap method, Table 4 evaluates the mediating role of physical activity intensity and time on the elderly's BMI. Combining the regression models and bootstrap analysis results indicated that physical activity acts as a mediator between the three dimensions of the built environment, including diversity, distance to public transportation, and destination accessibility aspects. Even though population density and road net density had significant effects on physical activity, the bootstrap analysis showed that no significant mediating effects of physical activity were found among population density, road net density, and BMI.

**Table 4:** Bootstrap results of mediating analysis.

**Mediating variables**	**Physical activity intensity**			**Physical activity duration**		
**BMI**	**Direct effects**	**Indirect effects**	**Total effects**	**Direct effects**	**Indirect effects**	**Total effects**
**Density**						
Population Density	0.0000	0.0000	0.0000	0.0000	0.0000	0.0000
**Diversity**						
Land-use mix	**0.3841**^**a**^ (92.33%)^b^	−0.1127^***^ (−27.09%)	0.4160	0.3841 (92.33%)	0.1446^***^ (34.76%)	0.4160
**Design**						
Road-net density	−0.0117 (90.70%)	−0.0006 (4.65%)	−0.0129	−0.0117 (90.70%)	−0.0006 (4.65%)	−0.0129
**Distance to transit**						
Bus stop	0.0045 (73.77%)	0.0002 (3.28%)	0.0061	0.0045 (73.77%)	0.0014^***^ (22.95%)	0.0061
**Destination accessibility**						
Hospital	−0.0443 (133.03%)	−0.0005 (1.50%)	−0.0333	−0.0443 (133.03%)	0.0115^***^ (−34.53%)	−0.0333
Park	0.0737^***^ (106.50%)	−0.0014^***^ (−2.02%)	0.0692^**^	0.0737^***^ (106.50%)	−0.0032^***^ (−4.62%)	0.0692^**^
Gym	−0.0044 (38.26%)	−0.0015^***^ (13.04%)	−0.0115	−0.0044 (38.26%)	−0.0056^***^ (48.70%)	−0.0115
Fast food restaurant	0.0050 (73.53%)	0.0022^***^ (32.35%)	0.0068^*^	0.0050 (73.53%)	−0.0004^***^ (−5.88%)	0.0068^*^

a *The coefficients of direct, indirect, and total effects*.

b *The proportion of direct and indirect effects in total effects*.

*The significant coefficients are in bold*.

*** *p < 0.001;*

** *p < 0.01;*

* *p < 0.05*.

First, the distance to public transportation and the number of hospitals were positively correlated with physical activity time; an increasing distance to the bus stops and more number of hospitals in the community caused elderlies to have less physical activity time and a higher BMI. Second, physical activity intensity and time had inverse effects on the relationship between land-use mix, fast-food restaurant accessibility, and BMI. The higher the land-use mix or the fewer the fast-food restaurants, the lower the elderly's physical activity intensity and the lower their BMI. However, higher land-use mix or fewer fast-food restaurants meant lower physical activity time and a corresponding increase in BMI for the residents, thus indicating that there may be no significant overall effect of land-use mix on the elderly's BMI. Third, gym accessibility can indirectly reduce BMI through both intensity and duration of physical activity. Lastly, park accessibility can directly increase the elderly's BMI levels. Park accessibility can also indirectly decrease the elderly's BMI levels by decreasing physical activity intensity and duration, with physical activity serving as a partial mediator.

## Discussion and Conclusion

Building upon the relationship between the built environment and residents' health outcomes, this study re-examined how physical activities in Shanghai mediate the association between the objective built environment and the elderly's BMI. We used the bootstrap method (20, 56) to investigate how the built environment affects overweight adults via physical activity mediators, including the traditional 5D built-environment variables (density, diversity, design, distance to transit, and destination accessibility). In summary, we discovered that physical activity time has a huge mediating effect on the elderly in Shanghai, particularly for the density and accessibility of facilities (gyms, parks, fast food restaurants) that can greatly stimulate physical activity to reduce BMI. Indeed, our findings add to the existing research in three ways:

First, we presented an additional set of physical activities that could partially or completely moderate the associations between home-based, objective built-environment qualities and the BMI of senior people (21, 43). We discovered that the built environment and its elements can promote two types of PA (activity intensity and activity time) concurrently. Built environment that supports physical activity (such as pedestrian-friendly streets and high accessibility to community activity facilities) can ensure that the elderly can perform high-quality PA in a safe and comfortable place. The time and intensity of PA will be affected by the built environment, which in turn affects the BMI index. However, activity intensity and activity duration had opposing effects on the elderly's BMI (Model 3), with activity time serving as a greater mediator between the built environment and BMI. One probable explanation is that aerobic and anaerobic workouts use different weight-loss systems (58).

Furthermore, we found a consistent results that calorie expenditure related facilities features are fully medicated by the physical activities (21). The effect of gym accessibility on BMI, for example, is largely mediated by physical activity duration (48.7%) and intensity (13.04%), respectively, whereas activity time mediated a 23% contribution of bus stop proximity on BMI. According to Frank et al. (25) and Saelens et al. (59), people living in a neighborhood with high population density, high land-use mix, and high connectivity have a lower BMI, but built-environment features related to walkability have been dropped in our model. Moreover, the mediation effect of PA also disappears on road network density and population density. One probable explanation is that the differences in built environments across communities in Shanghai's Yangpu District are not distinct enough. Furthermore, with the exception of Rutt and Coleman (26), we discovered that the influence of the degree of land mix on BMI can be mediated by activity intensity and duration. This, however, posed an opposite moderating effect. Increasing the land-use mix that led to the increase in walking for transportation was related to less moderate intensity and lower BMI. The elderly could benefit from less moderate intensity (such as walking or jogging) even when they were inactive for the majority of their lives. Less moderate intensity PA would still be related to increased energy expenditure and, theoretically, lower BMI for the elderly. However, a higher land-use mix resulted in less physical activity time and, as a result, an increase in BMI for the resident. It is well known that older people are among the most sedentary and physically inactive groups, with the increase in land use mix, residents' physical activity types changed, and the time of physical activity changed simultaneously. As Sallis et al. (14) stated that variations in built-environment types may result in inconsistent findings, and Noordzij et al. (60) stated the effect of mixed land use on physical activities such as cycling/walking is still unclear. In terms of calorie-related built environment, it has been discovered that food accessibility boosts senior people's BMI. It is possible to explain this by stating that cultural differences and eating habits cause inconsistencies in the influence of food accessibility on the BMI of the elderly. According to Raja et al. (61), when restaurants dominate non-residential land use, a diverse land-use mix in a community may be favorably related to BMI.

Third, we found that urban parks have both direct and indirect impacts on BMI, implying that the health benefit mechanism of green spaces for the elderly appears to be more complex (41). Many studies have now emphasized older people's sedentary behaviors in green spaces, such as resting, conversing, picnicking, and table gaming (62–64). According to Webster et al. (65), the elderly in the United Kingdom were more inclined to sit in parks rather than exercise, as more physical recreation occurred on the hard surface than in green space.

Our findings have far-reaching policy implications for the growth of health-focused communities in an aging city. According to this study, land-use mix, road network density, and accessibility to public facilities such as hospitals, parks and green spaces, and fitness centers, all have a substantial correlation with physical activity, influencing individual BMI via the mediating effect of physical activity. As a result, in community planning, it is recommended to avoid the development mode of super-large developments or to improve the provision of services and facilities for varying community purposes. Community activity centers, park green spaces, and so on provide facilities and places for older people to engage in physical activities in a comfortable and safe environment, as well as help promote healthy behavioral choices for outdoor activities. It is recommended that, in addition to improving parks and community activity centers to promote people's physical activity, more types of public activity spaces be developed and services be provided to carry out related activities, such as proposing public health-friendly space intensification models and developing the sharing potential of unused spaces.

## Limitations

There are two main limitations to this paper. Firstly, the measurement method for built-environment variables can be further optimized. For example, the measurement of park accessibility in this paper was measured as the number of parks in the buffer zone of the research object's residence, but the factors that affect the use of parks for the elderly also include the quality of greening, area, and facilities. In future research, more influence factors can be included, and the built environment can be measured more accurately and comprehensively.

Secondly, the Bootstrap method was used in this paper to examine the mediation effects between the built environment, physical activity, and BMI. Currently, it is recognized that the Bootstrap method directly tests the coefficient product ab, which is useful in various complex models. However, the bootstrap method also has limitations in that it may underestimate the direct impact effect (66). Moreover, since the research data is cross-sectional, the causal relationship between the urban built environment indicators and health outcomes cannot be proved. An environmental intervention should be tried in the future. Longitudinal data should be collected, and a model that can deal with multiple mediator variables, hierarchical data, and longitudinal data should be found to find out what caused the relationship.

## Data Availability Statement

The datasets presented in this study can be found in online repositories. The names of the repository/repositories and accession number(s) can be found in 2015 Shanghai Yangpu District Diabetes Health Records: http://www.scdc.sh.cn.

## Author Contributions

YX: writing—original draft, writing—review, and editing. SC: original draft and writing and formal analysis. SM: data analysis. YY: funding acquisition, validation, conceptualization, and supervision. All authors contributed to the article and approved the submitted version.

## Funding

This work was supported by The National Social Science Fund of China [No. 19BSH035], and The National Natural Science Foundation of China [No. 51878456].

## Conflict of Interest

The authors declare that the research was conducted in the absence of any commercial or financial relationships that could be construed as a potential conflict of interest.

## Publisher's Note

All claims expressed in this article are solely those of the authors and do not necessarily represent those of their affiliated organizations, or those of the publisher, the editors and the reviewers. Any product that may be evaluated in this article, or claim that may be made by its manufacturer, is not guaranteed or endorsed by the publisher.
